# Pseudo Foster-Kennedy Syndrome in an amblyopic patient: a case report

**DOI:** 10.22336/rjo.2025.38

**Published:** 2025

**Authors:** Cristina Ariadna Nicula, Oana Suluțiu

**Affiliations:** 1 Department of Maxillofacial Surgery and Radiology, “Iuliu Hațieganu”, University of Medicine and Pharmacy Cluj-Napoca, Romania; 2 County Emergency Hospital, Eye Clinic Cluj-Napoca, Romania

**Keywords:** pseudo Foster-Kennedy syndrome, amblyopia, non-arteritic anterior ischaemic optic neuropathy, NAION = non arteritic anterior ischaemic optic neuropathy, BCVA = best corrected visual acuity, RE = right eye, LE = left eye, ENT = ear, nose, throat, ESR = erythrocyte sedimentation rate, CRP = C reactive protein, GCA = giant cell arteritis

## Abstract

Pseudo-Foster-Kennedy syndrome presents with optic disc swelling in one eye and optic atrophy in the other eye. It differs from the true Foster-Kennedy syndrome due to the absence of an intracranial mass. One of the most common causes of pseudo-Foster-Kennedy syndrome is sequential bilateral NAION.

We present the case of a male patient who came to the emergency room in our clinic complaining of sudden vision loss in his right eye, headache, and hearing loss. The patient also had a diagnosis of high amblyopia in his left eye, where we also discovered an optic atrophy. We established the positive diagnosis of pseudo-Foster-Kennedy syndrome after a thorough anamnesis, ophthalmologic examination, and multiple investigations.

## Introduction

Non-arteritic anterior ischemic optic neuropathy is a common cause of vision loss in patients over 50 years old [1]. The ischemia of the optic nerve head determines swelling of the axons and, therefore, results in optic nerve head edema [2,3]. Risk factors are preponderant vascular (hypertension, hypercholesterolemia) and small discs (disc at risk). NAION presents with acute, painless vision loss and has a typical inferior altitudinal vision loss on visual field. Sequential bilateral NAION is one of the most common causes of pseudo-Foster-Kennedy syndrome [3,4].

## Case report

**Fig. 1A:**
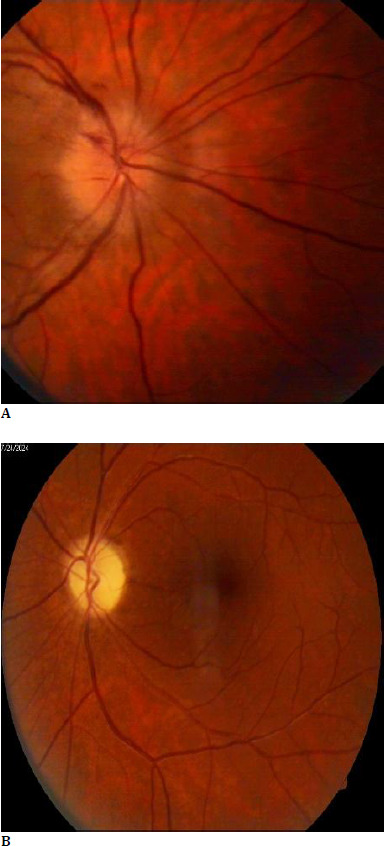
RE; B. LE

We present the case of a 61-year-old male patient who came to the emergency room in our clinic complaining of sudden vision loss in the right eye for 2 days, headache, and hearing loss. According to his medical history, the patient had a diagnosis of amblyopia (left eye). The ophthalmological examination revealed a BCVA in the right eye (RE) of 0.1 and in the left eye (LE) of 4/50. Autorefractometry readings for the right eye (RE) were: +1.00/-0.25 x 154 gr, and for the left eye (LE): +9.25/-3.00 x 28 gr. The intraocular pressure was normal in both eyes. Slit lamp examination revealed early cortical cataract, otherwise normal. At the RE fundus examination, we observed a swollen optic disc with multiple peripapillary hemorrhages and narrowing of the arteries (**Fig. 1A**). The LE fundus examination revealed a pale optic disc with the same narrowing of the arteries (**Fig. 1B**).

The requested blood tests, including a complete blood count, inflammatory markers, lipid panel, and hepatic and renal markers, were within normal limits. However, the glycemia levels were high at 142 mg/dl, but with a normal HbA1c. For the differential diagnosis, we also requested multiple immunologic markers (IgM and IgG antibodies for Borrelia Burgdorferi, Herpes simplex virus, Toxoplasma Gondii, and Toxocara canis), and all came back negative.

**Fig. 2A:**
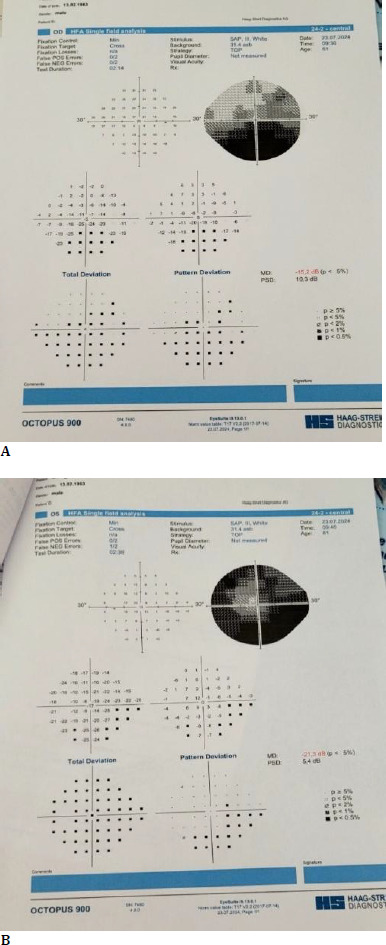
Inferior altitudinal defect in the right eye (RE); **B**. A narrowing of the visual field at 10°

Visual field testing revealed an inferior altitudinal defect in the right eye (RE) (**Fig. 2A**) and a narrowing of the visual field at 10° (**Fig. 2B**).

**Fig. 3:**
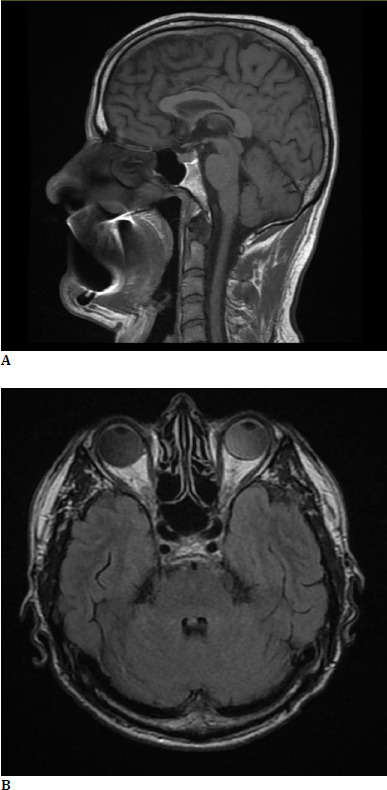
**A, B**. No cerebral lesions

Although our patient had a native cranio-cerebral CT scan (performed in the Emergency Room), which showed no signs of cerebral ischemia, cerebral hemorrhage, or cerebral tumors, we decided to request a cerebral MRI with contrast, which revealed an atrophy of the left optic nerve (~3 mm). No cerebral lesions were identified (**Fig. 3A, B**).

The neurologic examination did not reveal any neurologic pathology.

We requested a cardiologic consult, and the cardiologist diagnosed a stage II hypertension with very high additional cardiovascular risk. The carotid Doppler echography revealed a fibrocalcific atheromatous plaque in the right carotid and multiple fibrocalcific atheromatous plaques in the left carotid. The cardiologist recommended antiaggregant and antilipemic treatment, consisting of aspirin 75 mg (one tablet daily) and atorvastatin 20 mg (one tablet daily).

An ENT examination was requested, which revealed a bilateral neurosensory hearing loss, possibly of vascular origin.

Considering all of the above, we established the following positive diagnoses:

- RE: Non-arteritic anterior ischaemic optic neuropathy. Small hyperopia.
- LE: Optic atrophy (most likely vascular). Compound hyperopic astigmatism. High amblyopia. Anisometropia.
- Both eyes: Pseudo-Foster Kennedy Syndrome. Cortical cataract. Hypertensive arteriopathy.
- The differential diagnoses considered in this case were:
- Arteritic anterior ischemic optic neuropathy - associated with GCA, presents with headache, scalp pain, jaw claudication, and the optic nerve head has pale edema; ESR and CRP have high values [2,5].
- Optic neuritis - pain with eye movement, gradual vision loss, dyschromatopsia [6].
- Compressive lesions - progressive loss of vision, progressive exophthalmos, and limitation of the eye movements; easy to diagnose with CT scan or MRI [6].
- Other anterior optic neuropathies - nutritional (B12 or folic acid deficiency) or toxic (methanol) [5].
- Leber hereditary optic neuropathy - typically affects young patients but may also debut in older patients, initially affects one eye but becomes bilateral; the positive diagnosis is made with gene testing [5].

Without treatment, NAION evolves with low VA. With treatment, the VA may improve, and the complication rate lowers.

The treatment objectives for our patient were to reduce papillary edema and improve visual acuity (VA) in the right eye (RE). In the emergency room, a peribulbar injection with dexamethasone was performed in the RE (for the anti-edematous effect). Additionally, in the absence of ESR and CRP in the emergency department, the patient initiated general treatment with methylprednisolone 500 mg i.v., which was discontinued after 3 days. The patient received pentoxifylline 100 mg/5 ml, two tablets twice daily, aspirin 75 mg, one tablet once daily, atorvastatin 20 mg, one tablet once daily, and milgamma 100+100, one tablet once daily, upon discharge.

At the 6-week follow-up, the patient had a best-corrected visual acuity (BCVA) of 0.3 in the right eye (RE) and 4/50 in the left eye (LE). At the fundus examination of the RE, we observed a pale optic disc.

## Discussion

Mostly unilateral, NAION has a rate of approximately 15% at 5 years of affecting the other eye [6]. The presentation includes acute, painless vision loss with an inferior altitudinal defect in the visual field. The typical patient is over 50 years old with vascular risk factors and a small, crowded disc [1,3,7].

There is no specific treatment; the most crucial step is identifying the patients at risk and trying to control the risk factors. Corticosteroid use in patients with NAION may improve visual acuity (VA) and visual field defects at 6 months, possibly by enhancing local circulation and reducing edema. Aspirin, peripheral vasodilators, and neuroprotectors may be associated. Hyperbaric oxygen therapy and optic nerve sheath decompression have not been proven to be effective [2,6].

## Conclusions

NAION is a widespread pathology that affects patients over 50 years old. It is essential to differentiate from AAION (symptoms, signs, and ESR + CRP values). We can help our patients by identifying those who are at risk of developing NAION and by controlling the risk factors associated with it. Although we do not have a specific, targeted treatment for this disease, some treatments may stabilize or slightly improve VA.
